# Spatial Distribution of K13-Positive Airway Epithelial Cells in Idiopathic Pulmonary Fibrosis

**DOI:** 10.3390/biomedicines14030728

**Published:** 2026-03-23

**Authors:** Fei Teng, Qi Zheng, Yansong Bai, Qianqian Zhao, Yanghe Fu, Huiqi Dai, Chenwen Huang, Tao Ren

**Affiliations:** 1 Department of Respiratory and Clinical Care Medicine, Shanghai Sixth People’s Hospital Affiliated to Shanghai Jiao Tong University School of Medicine, Shanghai 200233, China; 2 Department of Pathology, Shanghai Sixth People’s Hospital Affiliated to Shanghai Jiao Tong University School of Medicine, Shanghai 200233, China; 3 Department of Clinical Research Centre, Shanghai Sixth People’s Hospital Affiliated to Shanghai Jiao Tong University School of Medicine, Shanghai 200233, China; 4 Stem Cell Center, Shanghai Sixth People’s Hospital Affiliated to Shanghai Jiao Tong University School of Medicine, Shanghai 200233, China

**Keywords:** idiopathic pulmonary fibrosis, KRT13, airway epithelial cell, proximalization, basal cell

## Abstract

**Background**: The progression of idiopathic pulmonary fibrosis (IPF) involves distal airway remodeling and bronchiolization; however, the mechanisms driving these changes, particularly the contributions of epithelial stem cells, are not fully understood. K13^+^ hillock cells, normally quiescent in proximal airways, were examined for their potential contribution to IPF pathogenesis. **Methods**: Spatial immunofluorescence was used to profile K13 expression along the airway axes in IPF and control lungs. Multiplex staining complemented by ex vivo culture assays was used to test expression stability. Single-cell RNA-sequencing (scRNA-seq) data were re-analyzed to identify cell subclusters and pathway enrichments. Meanwhile, cell–cell communication was inferred by using CellChat. **Results**: K13 was ectopically upregulated in IPF honeycomb cysts, triggering a proximal-like pseudostratified phenotype. This shift was marked by surges in K13^+^ regionally overlapping expression patterns (K5^+^, ~9%; CC10^+^, ~53%; ACE-TUB^+^, ~44%; MUC5AC^+^, ~23%) and a decline in SOX2 expression (~95% to ~64%), with ~70% of residual SOX2^low^ cells exhibiting elevated K13. Accompanying the expansion of K13^+^ subclusters (basal: 1.8% to 41.5%; club: 10.7% to 31.5%), it was observed that the profibrotic markers (K17, S100A2, LGALS7, IGFBP6) and ontologies related to RNA processing, stress response, and senescence were also enriched. These subclusters also amplified pro-fibrotic signaling (e.g., TGF-β, SEMA3, and GALECTIN-9) associated with epithelial subtypes and HAS1^high^ fibroblasts. **Conclusions**: Here, we demonstrate that K13^+^ cell activation is a pivotal event, driving the dysregulated proximalization of distal airways in IPF through fate reprogramming and epithelial-mesenchymal crosstalk. Thus, elucidating these K13-mediated fate dynamics provides a critical framework for understanding IPF pathogenesis.

## 1. Introduction

IPF is a relentlessly progressive fibrotic interstitial lung disease associated with a dismal prognosis, with a median survival of only 2.5 to 3 years [1,2]. The disease typically affects older adults and poses an increasing global health burden due to rising incidence rates, limited therapeutic options, and the lack of curative interventions other than lung transplantation [3,4]. Radiographic and histopathological examinations reveal some typical features in IPF, such as the patchy destruction of lung parenchyma and the presence of honeycomb cysts predominantly distributed in the subpleural and basal lung zones, reflecting advanced fibrotic remodeling and irreversible architectural distortion [5,6]. At the morphological level, these honeycomb lesions are accompanied by distinct structural aberrations of the distal airways, including an increased number of bronchiolar profiles, ectatic and tortuous lumens, thickened and disorganized walls, as well as widespread bronchiolization of the alveolar epithelium bordering fibrotic foci [7,8,9,10,11,12].

Emerging evidence over the past decade has shifted the paradigm of IPF pathogenesis from a predominantly inflammation-driven model toward one centered on aberrant epithelial–mesenchymal crosstalk and dysfunctions of endogenous epithelial stem/progenitor cells [13]. In particular, epithelial stem cells have been increasingly recognized as a key arbitrator of both injury repair and maladaptive remodeling [14]. Under homeostatic conditions, these epithelial stem cells initiate precise regeneration of the alveolar–bronchiolar niche following lung injury. However, in the context of IPF, recurring injuries to epithelium may provoke abnormal activation, incomplete differentiation, and persistent regeneration of these stem cells, thereby driving the progressive replacement of functional alveoli with fibrotic and cystically dilated bronchiolar-like structures [15,16,17,18]. Despite the conceptual advance implicating aberrant epithelial stem cell repair as a central pathogenic event underlying these structural abnormalities, the distinct contributions of individual epithelial stem cell subtypes to aberrant tissue remodeling and the precise mechanisms by which they propagate fibrogenesis are still largely undefined.

Among different airway epithelium stem cells, basal cells (BCs) play an indispensable role in maintaining epithelial integrity and conducting regenerative repair following lung injury [19,20,21]. Under pathological conditions such as IPF, persistent microinjuries and an increasingly dysfunctional niche are thought to disturb the normal regenerative program, inducing aberrant BC-mediated repair that contributes critically to the structural remodeling of the distal airways [22,23]. Recent advances in single-cell transcriptomics and high-resolution imaging have substantially refined our understanding of BC heterogeneity in the fibrotic lung. Compared to their counterparts in healthy individuals, BCs isolated from IPF distal lung tissue exhibit markedly expanded phenotypic diversity, along with newly emerging aberrant subpopulations [24]. These include K5^−^/K17^+^ basaloid cells, a population molecularly distinct from canonical basal stem cells and thought to represent an aberrant regenerative state [25,26,27,28]; CD66^+^ BCs and GPR87^+^ BCs, whose surface marker profiles suggest altered metabolic and signaling milieus [29,30]; and K8^+^ transitional stem cells, which poise at an intermediate differentiation stage yet fail to complete terminal maturation [31]. Functionally, these abnormal BC subpopulations universally exhibit diminished regenerative capacity, manifesting as cell cycle arrest, increased expression of senescence-associated markers, and a blunted response to mitogenic stimuli [32,33]. Concurrently, they acquire a pro-fibrotic phenotype characterized by the secretion of profibrotic cytokines, growth factors, and matrix-remodeling enzymes [32,34]. Through paracrine signaling, these senescent and functionally aberrant BCs promote the proliferation, activation, and myofibroblastic differentiation of lung fibroblasts, thereby driving excessive deposition and crosslinking of extracellular matrix (ECM) components [25,35,36]. This ECM accumulation first distorts alveolar architecture and thus creates a stiffened, pro-inflammatory niche that further exacerbates BC dysfunction and fuels a self-sustaining cycle of aberrant repair and fibrotic progression [37]. Together, these findings implicate the emergence of functionally aberrant, senescence-prone BC subpopulations as a central cellular mechanism underlying IPF progression.

In our study, we aimed to investigate the contribution of aberrant epithelial stem cell subpopulations to IPF progression within diseased lung regions and identify a previously unrecognized epithelial subpopulation K13^+^ epithelial cells that exhibits marked ectopic distribution in the distal airways and honeycomb lesions of IPF patients. Previous research has revealed that in healthy human lungs, K13^+^ epithelial cells are primarily confined to hillock structures within the cartilaginous airways, localized specifically along the axis of cartilage rings or at the cartilage-posterior membrane junction [38,39]. These cells have recently been identified as a transitional state along the basal-to-club cell differentiation trajectory and are therefore designated as K13^+^ hillock cells. Functionally, K13^+^ hillock cells resemble BCs in their capacity to participate in airway epithelial repair following injury. However, they possess markedly low proliferative activity and heightened resistance to damage, distinguishing them from regular BCs. Under homeostatic conditions, this population remains remarkably stable and serves to maintain hillock structural integrity even under severe injurious challenges. Usually, K13^+^ hillock cells are rarely detectable in the distal lungs of healthy individuals [39,40]. Surprisingly, in our experiment, we observed that K13^+^ epithelial cells ectopically emerge within inflammatory niche-enriched regions of IPF fibrotic foci, where they aberrantly express multiple markers of epithelial differentiation, including MUC5AC, CC10, and ACE-TUB, a feature not typically associated with their hillock-restricted identity. This ectopic appearance and aberrant differentiation phenotype suggest that K13^+^ epithelial cells may participate in dysregulated airway repair and contribute to the structural remodeling of the distal fibrotic lung. More broadly, these findings reveal an emerging pathological paradigm in IPF: the distal lung epithelium acquires a proximal airway-like molecular and cellular identity, a process we define as “distal airway proximalization”, which serves as a key driver of honeycomb cyst formation and progressive alveolar functional decline.

## 2. Materials and Methods

### 2.1. Human Lung Specimens

All human tissue samples used in this study were obtained and handled in accordance with the research protocol approved by the Ethics Committee of Shanghai Jiao Tong University Affiliated Sixth People’s Hospital (Approval No. 2025-KY-259(K)), and all relevant guidelines and regulations were followed. Written informed consent was obtained from all participants prior to sample collection. Lung tissues for pathological analysis were collected from explanted lungs of patients with end-stage IPF who underwent lung transplantation. IPF was diagnosed based on the consensus criteria of the American Thoracic Society and European Respiratory Society [41]. Fibrotic regions were identified preoperatively using high-resolution computed tomography (HRCT), and multiple representative lesional areas were sampled. Control lung tissues were obtained from patients undergoing lobectomy for solitary pulmonary nodules. Non-neoplastic lung parenchyma located distant from the nodule and confirmed as histologically normal by the Department of Pathology was used as control tissue. All sample collection procedures were approved by the Ethics Committee and conducted in full compliance with institutional and legal requirements.

### 2.2. Hematoxylin-Eosin (HE) Staining

Post-mortem lung samples were fixed in 4% formaldehyde solution (Servicebio, Wuhan, China, G1101) and subsequently embedded in paraffin. Tissue sections of 5 μm thickness were mounted on glass slides, followed by sequential deparaffinization with xylene and graded ethanol solutions (100%, 90%, 80%, and 70%). The sections were then stained with hematoxylin for 10 min, followed by three washes in PBS and a rinse in distilled water. The slides were then stained with eosin for 1 min (Beyotime, Shanghai, China, C0105S), and washed twice with 70% ethanol. Dehydration was performed through graded ethanol solutions (70%, 80%, 90%, and 100%), followed by clearing in xylene for 5 min. Finally, the sections were mounted with neutral resin (Beyotime, Shanghai, China, C0173) and allowed to dry. After confirming the quality of the slides under a microscope, a KF-PRO-120 digital slide scanner was used for rapid whole-slide scanning. Observations and analysis were performed using the KFSlideOS (v1.1.2.0) fully automated digital slide viewing system.

### 2.3. Immunofluorescence Staining of Lung Pathological Slices

For each anatomical region described above, three independent sections were prepared for immunofluorescence staining. After dehydration, the sections were incubated in diluted sodium citrate antigen retrieval solution (Beyotime, Shanghai, China, P0081) and subjected to microwave heating for 20 min. After natural cooling, the sections were washed in PBS and permeabilized with 0.1% Triton X-100 (Beyotime, Shanghai, China, ST1723) for 30 min, followed by further washing and blocking for 30 min. Primary antibodies suitable for human samples were applied overnight at 4 °C, including rabbit anti-KRT13 (1:500, Abcam, Cambridge, UK, ab92551), rabbit anti-KRT5-Alexa Fluor 488 conjugated (1:500, Abcam, Cambridge, UK, ab193894), rabbit anti-KRT5 (1:100, Abcam, Cambridge, UK, ab52635), mouse anti-KRT5 (1:100, Thermo Fisher Scientific, Shanghai, China, MA5-17057), rabbit anti-Uteroglobin (CC10) (1:200, Abcam, Cambridge, UK, ab307666), mouse anti-ACE-TUB (1:150, Sigma-Aldrich, MA, USA, T7451), rabbit anti-MUC5AC (1:250, Abcam, Cambridge, UK, ab198294), and mouse anti-SOX2 (1:100, Thermo Fisher Scientific, Shanghai, China, MA1-014), and rabbit anti-Ki67 (1:250, Thermo Fisher Scientific, Shanghai, China, MA5-14520). On the following day, after washing, secondary antibodies were applied for 1 h at room temperature, including goat anti-rabbit Alexa Fluor 568 (1:500, Thermo Fisher Scientific, Shanghai, China, A-11011), goat anti-mouse Alexa Fluor 555 (1:500, Abcam, Cambridge, UK, ab150114), goat anti-rabbit Alexa Fluor 488 (1:500, Abcam, Cambridge, UK, ab300671), goat anti-mouse Alexa Fluor 488 (1:500, Abcam, Cambridge, UK, ab150113), donkey anti-rabbit Alexa Fluor 647 (1:500, Abcam, Cambridge, UK, ab150075), and donkey anti-mouse Alexa Fluor 647 (1:500, Abcam, Cambridge, UK, ab150107). For samples with primary antibody species conflicts or triple labeling, a multiplex fluorescence reagent kit (AFIHC023 and AFIHC024, Hunan AIFang Biological, Changsha, China) was used according to the manufacturer’s instructions. After washing, sections were mounted using an anti-fade mounting medium containing 4,6-diamidino-2-phenylindole (DAPI) (Servicebio, Wuhan, China, G1407). After the slides were air-dried, panoramic fluorescence scanning was performed using the 3DHISTECH Pannoramic MIDI digital slide scanner (Budapest, Hungary). Observations and analyses were conducted using SlideViewer (v2.4), and ImageJ (v1.51) (National Institutes of Health, Bethesda, MD, USA) was employed to measure fluorescence intensity and quantitatively analyze the co-expression of fluorescence signals.

To ensure an objective and unbiased definition of regions of interest (ROIs) and individual cells, this study employed an automated software-based segmentation approach. High-resolution confocal images were first preprocessed to subtract background and normalize intensity levels. Cell boundaries and labeled signal regions were then segmented using automatic thresholding algorithms, followed by the application of the watershed algorithm to separate touching or overlapping cells. For unbiased sampling, multiple fields of view were systematically acquired across airway regions of entire tissue sections and defined as ROIs. Prior to analysis, individual fluorescence channels were digitally separated using SlideViewer image browsing software. Quantitative co-localization analysis was performed with the Colocalization Finder plugin (v1.3) in ImageJ (v1.51). For each image, intensity thresholds for each channel were set independently based on the signal distribution in the corresponding fluorescence histogram. The lower threshold was conservatively positioned at the inflection point marking the boundary between specific signal and non-specific background fluorescence, thereby minimizing the impact of background noise on co-localization measurements. Co-localization was quantified using Manders’ overlap coefficients M1 and M2, where M1 represents the fraction of signal in channel 1 that overlaps with signal in channel 2, and M2 the reverse. These coefficients are independent of differences in signal intensity ratios between channels. Co-localization was considered biologically meaningful when M-values exceeded 0.5 [42].

### 2.4. Preparation, Cultivation, and Passage of Human BCs

Airway epithelial cell isolation and basal cell culture were performed as previously described [43,44]. Proximal airway epithelial stem cells were obtained by bronchial brushing. Distal airway epithelial cells were isolated by enzymatic digestion of explanted lungs from patients with end-stage IPF undergoing lung transplantation. Following isolation, all cells were expanded in PneumaCult Ex Plus Medium (Stemcell Technologies, Vancouver, BC, Canada) at 37 °C in 5% CO_2_. The medium was changed every two days, and cells were passaged at a ratio of 1:1 to 1:2 upon reaching 80–90% confluence.

### 2.5. Flow Cytometry Sorting (FACS)

Primary epithelial stem cells, isolated from bronchoalveolar lavage fluid (BALF) and expanded in culture, were transduced with an LV3 lentiviral vector encoding a KRT13-reporter-GFP construct. Following 72 h of incubation to permit transgene expression, cells were harvested and sorted by fluorescence-activated cell sorting (FACS) on a MoFlo XDP cell sorter (Beckman Coulter, Brea, CA, USA). GFP-positive cells were gated based on green fluorescence (excitation: 488 nm laser; emission: 530/40 nm bandpass filter) and collected into sterile tubes for downstream analyses.

### 2.6. ELISA Assay

We measured TGF-β levels in culture supernatants harvested from FACS-sorted K13^+^ and K13^−^ cells. Supernatants were collected separately from K13^+^ and K13^−^ cell cultures. TGF-β concentrations were quantified using a commercial TGF-β ELISA kit (Abcam, Cambridge, UK, ab108912) in accordance with the manufacturer’s instructions.

### 2.7. Cell Immunofluorescence Staining

Cells were fixed with formaldehyde (Servicebio, G1101) for 10 min, followed by washing with PBS. The cells were then permeabilized with 0.2% Triton-X (Beyotime, Shanghai, China, ST1723) for 20 min and washed with PBS. After permeabilization, the cells were blocked with PBST (0.05% Tween-20, 5% FBS) for 1 h. The cells were then incubated overnight at 4 °C with primary antibody rabbit anti-KRT13 (1:500, Abcam, Cambridge, UK, ab92551), rabbit anti-KRT5-Alexa Fluor 488 conjugated (1:500, Abcam, Cambridge, UK, ab193894), and rabbit anti-Ki67 (1:250, Thermo Fisher Scientific, Shanghai, China, MA5-14520). After washing with PBS, the cells were incubated for 1 h in the dark with secondary antibody goat anti-rabbit Alexa Fluor 568 (1:500, Thermo Fisher Scientific, Shanghai, China, A-11011), and donkey anti-rabbit Alexa Fluor 647 (1:500, Abcam, Cambridge, UK, ab150075). After further washing, DAPI (1:500, Sigma-Aldrich, MA, USA) was applied to stain the nuclei for 10 min. Images were captured using an Olympus FV1200 scanning confocal microscope and then processed and analyzed using FV10-ASW 4.2 Viewer (Tokyo, Japan) and ImageJ (v1.51).

### 2.8. ScRNA-Seq Data Analysis

Systematic analysis of the GSE135893 dataset from the Gene Expression Omnibus (GEO) database was performed using Seurat (v5.0.5) [28]. The cell expression matrix was loaded and converted into a Seurat object, followed by initial quality control. Genes expressed in fewer than 200 cells and cells expressing fewer than 3 genes were filtered out. Quality control was also performed based on the proportion of mitochondrial genes, gene count, and UMI count. Specifically, cells with more than 10% of transcripts originating from mitochondrial genes were excluded. Additionally, cells with nFeature_RNA < 300 or >5000, or nCount_RNA < 500 or >15,000, were filtered out to ensure data quality. The data were log-transformed using global scaling normalization, and the NormalizeData function with default parameters was used for standardization and normalization of the data. Next, the FindVariableFeatures function was employed to identify the top 2000 highly variable genes, which exhibited the greatest expression variability across cells and were selected for downstream analysis. Data were linearly scaled using the ScaleData function to center the transcriptomic data to a mean of 0 and a variance of 1, thereby removing the influence of technical factors (such as sequencing depth). Principal component analysis (PCA) was then performed on the highly variable genes, using the RunPCA function to identify the top 50 principal components, followed by the ElbowPlot function to determine the number of significant principal components.

K-nearest neighbor graphs were constructed using the FindNeighbors function (dims = 1:30), and optimal resolution for cell clustering was determined using the FindClusters function, applying the Louvain algorithm with a resolution of 0.5. Subsequently, non-linear dimensionality reduction was performed using the RunUMAP function (dims = 1:30) to generate a UMAP plot. Subpopulations of epithelial cells and fibroblasts were annotated using metadata provided in a previously published study [31]. The expression matrix from the Seurat object was extracted, and the K13 gene expression column was isolated, and this result was added to the Metadata dataframe.

### 2.9. Stemness Score and Senescence Score

Stemness score was assessed using the CytoTrace2 R package (v1.0.0) to analyze cell developmental potential and cell potency categories from scRNA-seq data. For this study, the stemness score was based on gene expressions according to previously published protocols [45,46]. CytoTrace2 is a specialized algorithm tool based on gene expression profiles, designed to reveal the differentiation process of cells from scRNA-seq data.

The senescence score was calculated using the Python (v3.11) package SenePY [47]. This method innovatively leverages the biological principle that “senescence cells accumulate with age and remain in a small fraction,” by constructing gene networks to identify senescence features specific to cell types. The evaluation was conducted using the senescence gene signature reported in previous studies [48].

### 2.10. Cell Communication Analysis

Cell communication was analyzed using CellChat (v1.1.3). As the core step of inferring the cell communication network [49], the algorithm integrates normalized gene expression data with a ligand-receptor database. The interaction strength is evaluated by calculating the truncated mean, and statistical significance is assessed using a permutation testing method. Subsequently, the probability and strength of interactions between different cell types are calculated, enabling the analysis and visualization of communication patterns.

### 2.11. Statistical Analysis

All statistical analyses were performed using GraphPad Prism (v10.0). Quantitative data were obtained from at least three independent experimental samples and are presented as the mean ± standard deviation (SD). For comparisons between multiple groups, one-way ANOVA was used, and statistical significance was defined as *p* < 0.05. Statistical significance is indicated by asterisks as follows: * *p* < 0.05; ** *p* < 0.01; *** *p* < 0.001; **** *p* < 0.0001, while NS denotes no statistical significance (*p* > 0.05).

## 3. Results

### 3.1. Landscape of K13^+^ Cells Along the Proximal–Distal Axis of Airways in IPF

**Figure 1:**
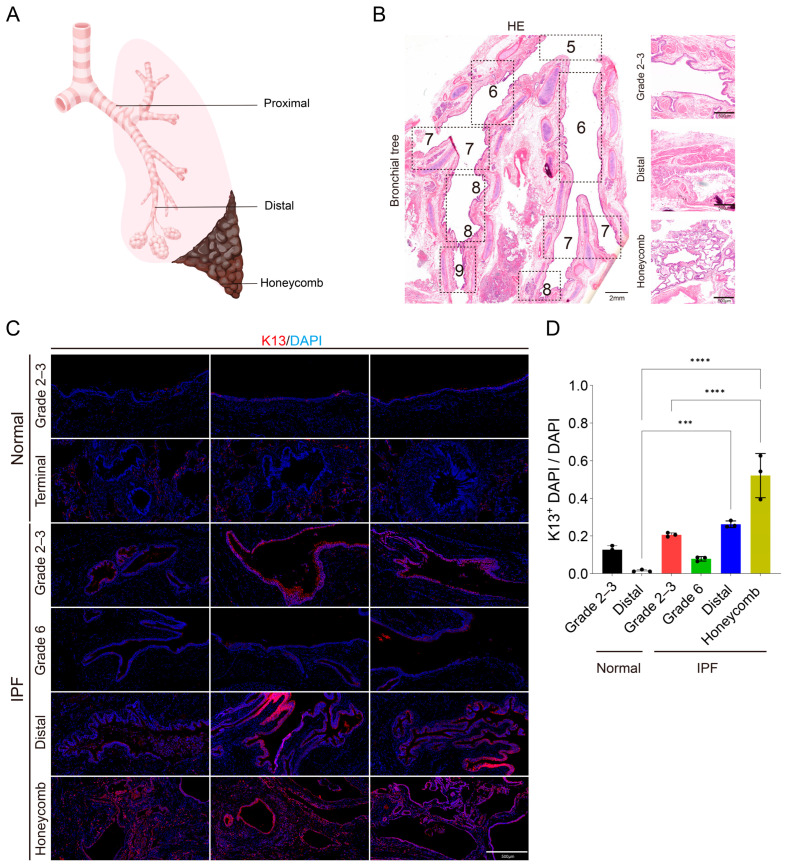
Landscape of K13^+^ cells along the proximal–distal axis of airways in IPF lung and normal control. (**A**) Diagram illustrating lung tissue sampling sites for IPF and normal control. Samples are collected from the proximal airway, distal airway, and subpleural lung edges. (**B**) HE staining of tracheal longitudinal sections from IPF lungs, including Grade 5–9 bronchial tree (left), Grade 2–3 (upper right), distal airway (middle right), and honeycomb (lower right). (**C**,**D**) Immunofluorescence staining for K13 (red) in airway tissue sections from normal lungs and three IPF patients (N = 3), along with corresponding statistical analysis of K13^+^ cell proportions. One-way ANOVA statistical analysis. *** indicates *p* < 0.001, **** indicates *p* < 0.0001.

In this study, we systematically examined the expression of K13 along the proximal-to-distal axis of the airways, as well as in the characteristic terminal honeycomb lung lesions of IPF, using lung tissue specimens from IPF patients (Supplementary Table S1) with lung transplantation. For comparison, non-fibrotic normal lung tissues surgically resected from non-IPF patients served as controls. Consistent with previous reports, IPF honeycomb lungs exhibited distinct airway remodeling features, characterized by aberrant bronchiolar dilation and prominent bronchiolar metaplasia of the distal alveolar epithelium (Figure 1A,B; Supplementary Figure S1A). To further delineate the spatial distribution of K13^+^ cells within the IPF airway epithelium, we performed K13 immunofluorescence staining across different airway segments. Compared to control lung tissues, K13 expression was significantly elevated in the distal airways of IPF lungs and displayed marked heterogeneity across various airway levels (Figure 1C,D; Supplementary Figures S1B and S2A). Notably, characteristic high K13 expression was also observed in subpleural honeycomb lesions, whereas K13 signals were virtually undetectable in normal distal subpleural lung parenchyma.

To further assess site-specific differences in K13 expression among airway epithelial stem cells derived from different anatomical sites, we isolated cells from three sources: proximal airways (via bronchial brushing), distal airways (from explanted IPF lungs), and the pathological microenvironment (from bronchoalveolar lavage fluid, BALF). Following in vitro culture, the proportion of K13^+^ epithelial stem cells was significantly higher in distal lung-derived and BALF-derived cultures compared to those derived from proximal airways (Supplementary Figure S2B,C). We further sorted K13^+^ and K13^−^ epithelial stem cells from BALF by flow cytometry and cultured them separately. Although no significant differences were observed in K5 or KI67 expression between the two populations, K13^+^ cells secreted significantly higher levels of TGF-β (Supplementary Figure S2D–G). This suggests that K13^+^ epithelial stem cells may contribute to pulmonary fibrosis progression through paracrine TGF-β signaling.

### 3.2. Identity and Plasticity of K13^+^ Epithelial Populations in IPF Airways

**Figure 2:**
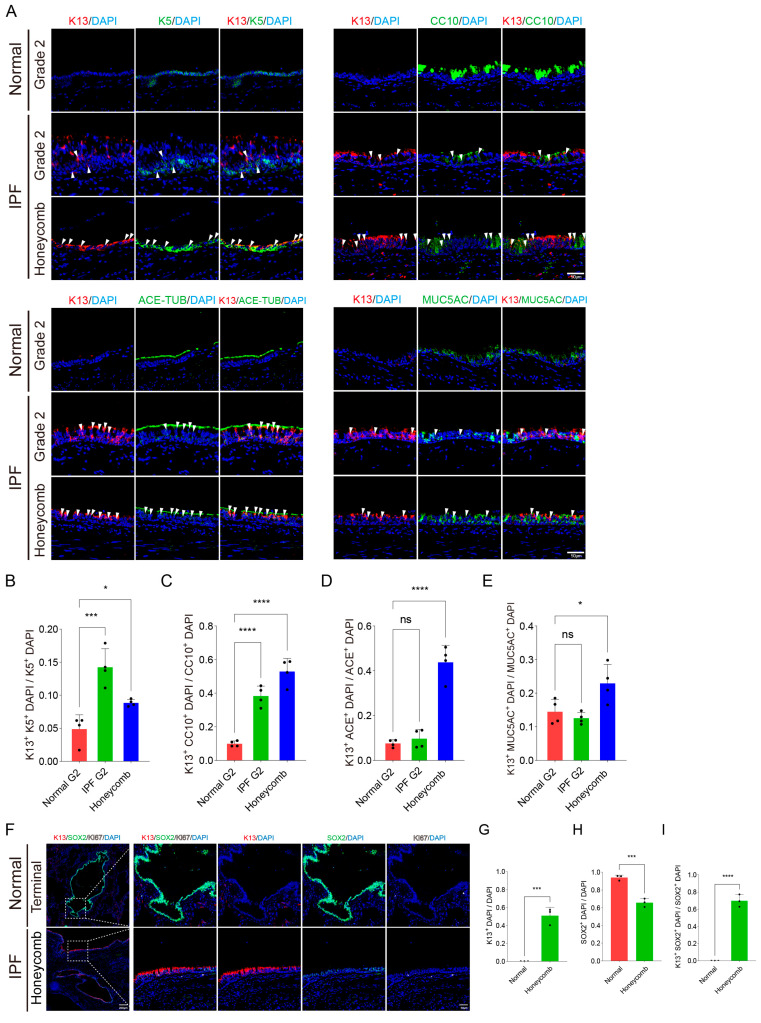
Characterization of K13^+^ cells in IPF lung and normal control. (**A**) Co-staining of K13 (red) and airway epithelial lineage cell markers (K5, CC10, ACE-TUB, and MUC5AC) (green) in the proximal airway of normal and IPF lung (N = 4 patients). The results are presented separately for each individual channel (K13 and each marker) and for the merged channels to visualize the co-expression of K13 with the airway epithelial markers. White arrowheads indicate cells exhibiting adjacent or regionally overlapping expression patterns. (**B**–**E**) Statistical analysis of the proportion of K13^+^ cells among various epithelial lineage cell populations was performed. (**F**) Representative pathological sections (N = 3 patients) of K13 (red) and SOX2 (green) co-staining from the normal terminal peripheral lung and IPF honeycomb. (**G**–**I**) Corresponding statistical analysis of the proportion of K13^+^ cells, SOX2^+^ cells, and the proportion of K13^+^SOX2^+^ co-localized cells among SOX2^+^ cells. One-way ANOVA statistical analysis means ± SD. * indicates *p* < 0.05, *** indicates *p* < 0.001, **** indicates *p* < 0.0001, while ns denotes no statistical significance (*p* > 0.05).

To characterize the specific subpopulations of K13^+^ cells, we performed multiplex immunofluorescence co-expression analysis of K13 with multiple airway epithelial lineage markers. Compared to healthy controls, IPF proximal airways showed a significant increase in the proportions of K5^+^K13^+^ and CC10^+^K13^+^ adjacent overlapping expression patterns (Figure 2A,B). Notably, in distal honeycomb lung regions, beyond marked increases in these two populations exhibiting adjacent or regionally overlapping expression patterns, we also observed the emergence of ACE-TUB^+^K13^+^ and MUC5AC^+^K13^+^ cell subsets displaying regionally overlapping expression patterns, which were virtually absent in normal distal airways(Figure 2A–E; Supplementary Figure S2H). By examining the expression of SOX2, a marker of airway epithelial stemness [50], we determined that although the total number of SOX2^+^ cells was significantly reduced in IPF distal lung tissues, the proportion of SOX2^+^ cells co-expressing K13 showed a marked increase (Figure 2F–I). This finding suggests that K13^+^SOX2^+^ cells may represent a critical intermediate transitional state of epithelial stem cells during lung structural remodeling.

### 3.3. Single-Cell Analysis Reveals the Expansion and Heterogeneity of K13^+^ Epithelial Cells in IPF

**Figure 3:**
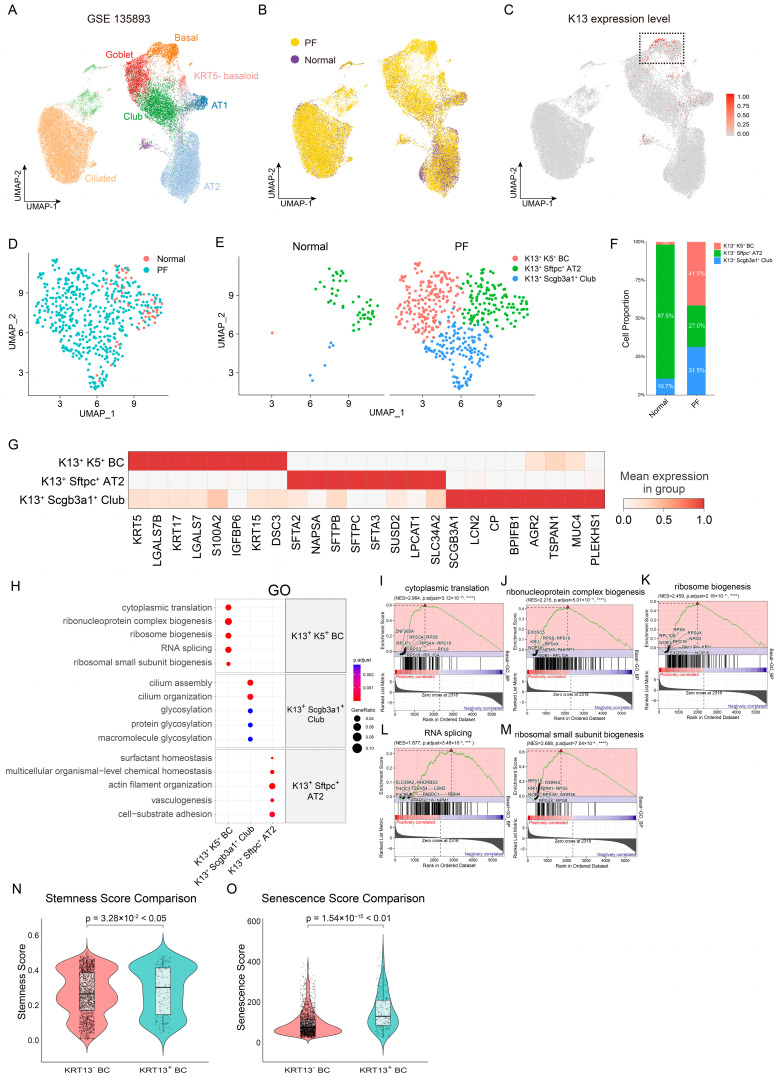
scRNA-seq of K13^+^ cells in the distal lung of IPF and normal control. (**A**–**C**) UMAP dimensionality reduction in epithelial cell clusters from the scRNA sequencing dataset GSE135893. (**D**–**F**) Re-clustering of the K13^+^ epithelial stem cell population in this dataset using UMAP. Panel (**D**) was split based on disease status (**E**), and the proportion of each cluster was quantified (**F**). (**G**) The heatmap shows markers across clusters, displaying the expression of 24 top markers. (**H**) GO term enrichment analysis of biological processes was performed using the FindAllMarkers function in Seurat. The color represents the adjusted *p*-value, and the size of the dots indicates the gene ratio. (**I**–**M**) GSEA analysis of the scRNAseq dataset. A positive enrichment score on the *y*-axis indicates a positive correlation with the K13^+^K5^+^ BC group, and a negative enrichment score indicates a negative correlation. (**N**,**O**) Violin plot comparing stemness (**N**) and senescence scores (**O**) between K13^−^ BC and K13^+^ BC. *** indicates *p* < 0.001, **** indicates *p* < 0.0001.

To investigate the molecular characteristics and potential pathogenic roles of K13^+^ cells in the distal lung and honeycomb lesions of IPF patients, we re-analyzed a previously published single-cell transcriptomic dataset GSE135893 derived from marginal lung tissues of IPF patients and healthy controls (Figure 3A,B). UMAP dimensionality reduction revealed that K13^+^ cells were markedly increased in IPF samples compared to healthy controls and were predominantly enriched within the basal cell cluster (Figure 3C; Supplementary Figure S3). Subclustering analysis of K13^+^ epithelial stem cells, including BCs, Club cells, and AT2 cells, showed that the proportions of K13^+^ Club cells and K13^+^ BCs within the total K13^+^ epithelial stem cell population were significantly elevated in IPF (Figure 3D,E). Specifically, K13^+^K5^+^ BCs increased from 1.8% in healthy controls to 41.5% in IPF, while K13^+^SCGB3A1^+^ Club cells rose from 10.7% to 31.5% (Figure 3F). To further confirm that the K13-positive signal was not attributable to random technical artifacts, we examined its expression patterns across major cell types in the tissue. The analysis showed that K13 expression was almost exclusively confined to epithelial cells, with minimal to undetectable levels in immune cells, stromal cells, and endothelial cells (Supplementary Figure S4). To validate the robustness of this cell-type-specific pattern, we re-analyzed an independent single-cell RNA-seq dataset (GSE227136) [51]. The results recapitulated our primary findings, confirming consistent K13 enrichment in epithelial populations (Supplementary Figure S5).

Gene expression profiling revealed that IPF-derived K13^+^K5^+^ BCs significantly upregulated the expression of K17 (an aberrant basal cell marker) [25] and the pro-fibrotic gene S100A2. In contrast, K13^+^SCGB3A1^+^ Club cells showed high expression of LCN2 (associated with poor IPF prognosis) [52,53], MUC1 (the mucin synthesis-related gene), and AGR2 (a regulator of epithelial differentiation) [54] (Figure 3G). GO enrichment and GSEA analysis indicated that K13^+^K5^+^ BCs were significantly enriched in pathways related to nuclear RNA processing, cytoplasmic translation, and ribosome biogenesis, suggesting their role in post-transcriptional modification and protein synthesis (Figure 3H–M). To further characterize the functional status of K13^+^K5^+^ BCs, we performed stemness and senescence scoring using stem cell signature gene sets and senescence-associated gene sets, respectively (Figure 3N,O). Unexpectedly, compared to K13^−^ BCs, K13^+^K5^+^ BCs exhibited both higher stemness potential and elevated senescence scores. These findings suggest that this subpopulation may represent an intermediate epithelial stem cell subset characterized by concurrent high stemness potential and accelerated senescence, reflecting a disease microenvironment-induced transitional state during stem cell differentiation.

### 3.4. Enhanced Epithelial Signaling in K13^+^ BCs and AT2 Cells Relative to K13^−^ Counterparts in IPF

**Figure 4:**
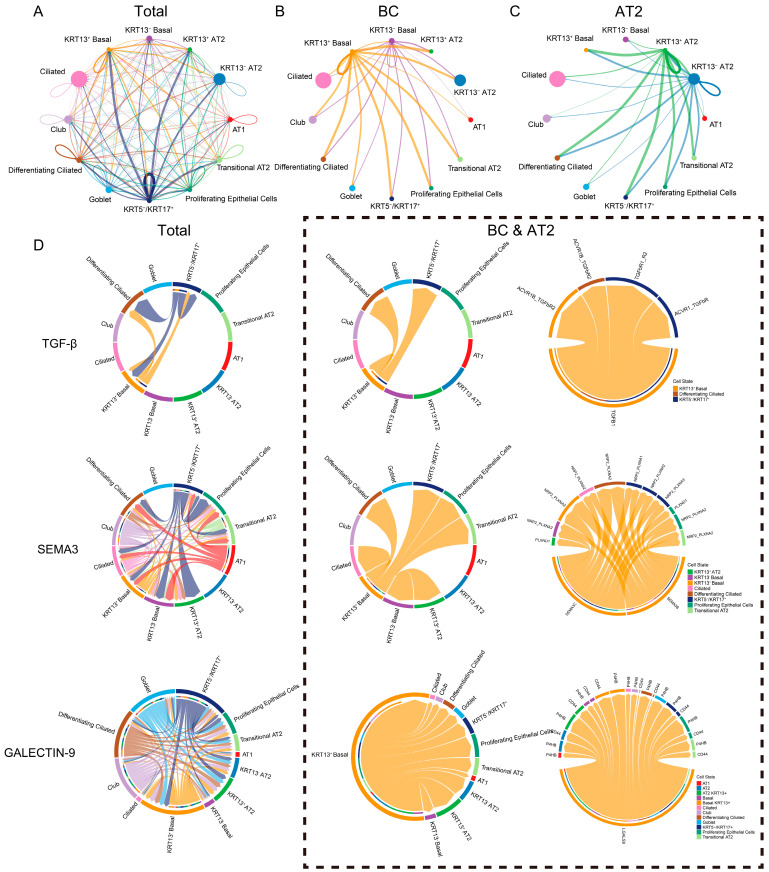
K13^+^ BCs and AT2 Cells Exhibit Stronger Communication with Epithelial Cells Compared to K13^−^ Cells. (**A**–**C**) CellChat analysis was performed to specifically characterize the intercellular signaling interactions between K13^+^ BCs, K13^+^ AT2 cells, and other lung epithelial cell clusters. Circle plots showing the overall cell–cell communication network strength (**A**), with separate CellChat results for K13^+^ BCs (**B**) and K13^+^ AT2 cells (**C**). (**D**) Illustrate the intercellular signaling interactions between K13^+^ BCs and K13^+^ AT2 cells and other epithelial cells through the TGF-β, SEMA3, and GALECTIN-9 signaling pathways. The left-to-right panels represent overall cluster communication, isolated communication when K13^+^ BCs and AT2 cells act as ligand cells, and detailed ligand–receptor communication within each pathway.

To investigate the potential interactions between K13^+^ epithelial stem cells and other epithelial cell types, we systematically analyzed cellular communication networks using CellChat. Our analysis revealed that, compared to their K13^−^ counterparts, K13^+^ BCs and AT2 cells exhibited more active intercellular communication (Figure 4A–C). To identify the specific signaling pathways responsible for this enhanced communication, we performed detailed ligand–receptor interaction analysis between K13^+^ BCs/AT2 cells and other airway epithelial populations (Supplementary Figure S6A,B). Within the epithelial communication network, K13^+^ BCs displayed a more prominent role in ligand-sending than K13^−^ BCs. Notably, ligands of the TGF-β signaling pathway (a key pro-fibrotic cascade) were highly expressed in K13^+^ BCs and showed strong communication with K5^+^K17^+^ basaloid cells (Figure 4D). In addition, other signaling pathways related to fibrosis progression and inflammation regulation, including SEMA3 [55,56], GALECTIN-9 [57], and THBS [58,59], exhibited significantly enhanced interaction strength in K13^+^ BCs compared to K13^−^ BCs (Figure 4D; Supplementary Figure S7).

### 3.5. Enhanced Communication of K13^+^ BCs and AT2 Cells with HAS1^high^ Fibroblasts in IPF

**Figure 5:**
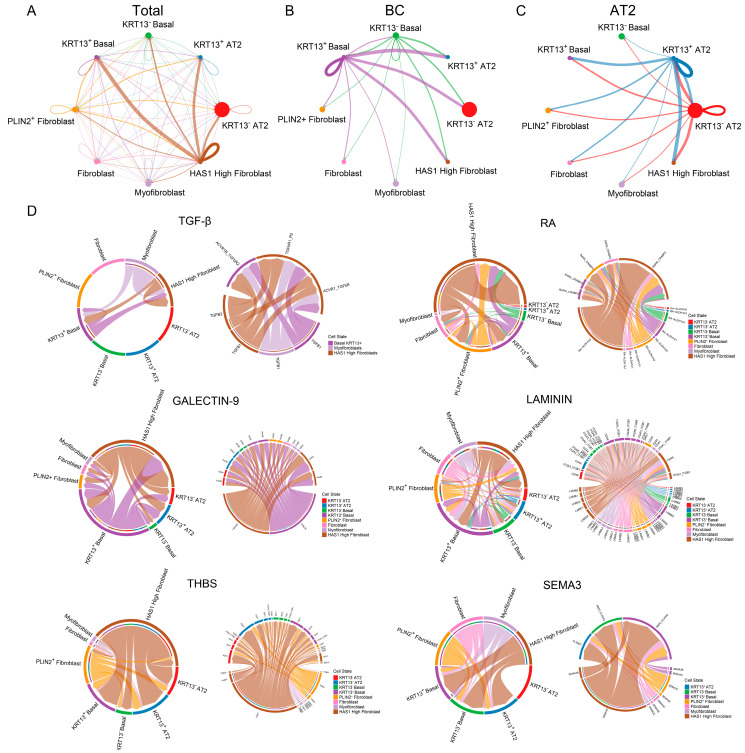
K13^+^ BCs and AT2 Cells Exhibit Stronger Communication with HAS1^high^ Fibroblasts. (**A**–**C**) CellChat analysis specifically characterizes the intercellular interactions between K13^+^ BCs, K13^+^ AT2 cells, and fibroblast subtypes. Circle plots display the overall strength of cell-cell communication (**A**), with separately showing the CellChat results for K13^+^ BCs (**B**) and AT2 cells (**C**). (**D**) Illustrate the cell-cell communication between K13^+^ BCs and AT2 cells and fibroblast subtypes through TGF-β, RA, GALECTIN-9 LAMININ, THBS and SEMA3 signaling pathways. The right panel provides a detailed view of the ligand-receptor interactions within these pathways.

We further explore the potential interactions between K13^+^ epithelial stem cells and stromal fibroblasts by fully analyzing cellular communication networks using CellChat. The result showed that K13^+^ BCs and AT2 cells exhibited more active intercellular communication with fibroblasts compared to their K13^−^ counterparts, with the most pronounced interactions observed with HAS1^high^ fibroblasts (Figure 5A–C). HAS1^high^ fibroblasts represent a distinct activated fibroblast subpopulation characteristically enriched in the subpleural regions (honeycomb lesions) of IPF lungs and may contribute to the accumulation of cellular stress and inflammatory mediators such as IL-4/IL-13 [28,60,61]. To further dissect the specific signaling pathways underlying this enhanced communication, we performed detailed ligand–receptor interaction analysis between K13^+^ BCs/AT2 cells and distinct fibroblast subpopulations (Supplementary Figure S8A,B). Within the TGF-β signaling pathway, K13^+^ BCs functioned both as major signal recipients that receive TGF-β signals from pro-fibrotic myofibroblasts and HAS1^high^ fibroblasts, and as ligand-sending cells, transmitting signals to HAS1^high^ fibroblasts. In addition, K13^+^ BCs served as key ligand donors in intercellular communication mediated by THBS, SEMA3, and LAMININ signaling pathways (Figure 5D). Collectively, these findings suggest that K13^+^ epithelial stem cells and pro-fibrotic fibroblasts may actively engage in reciprocal crosstalk, together forming a distinct injury-associated epithelial–mesenchymal interaction network.

## 4. Discussion

This study reveals, for the first time, a significant ectopic expression of K13 in the lung tissue of IPF patients. Notably, a marked accumulation of K13^+^ cells was observed within honeycomb cysts, particularly in the subpleural regions. To further characterize the phenotype of these K13^+^ cells in IPF, we performed multiplex immunofluorescence staining. Our analysis revealed not only a significant increase in the proportion of K13^+^K5^+^ and K13^+^CC10^+^ cells exhibiting adjacent or regionally overlapping expression patterns in the distal airways and honeycomb lesions, but also the emergence of K13^+^ACE-TUB^+^ and K13^+^MUC5AC^+^ cell populations displaying regionally overlapping expression patterns, which are exceptionally rare in normal airways. Collectively, these results suggest that, within the pathological context of IPF honeycomb lung, the identity of distal airway epithelial cells shifts toward a proximal airway epithelial cell phenotype. The ectopic expression of K13 indicates that the reprogrammed epithelial cells in IPF possibly arise from two distinct origins. First, normally quiescent K13^+^ hillock cells may serve as a stem cell reservoir. Under pathological conditions, these cells could migrate to the distal lung to repopulate depleted stem cell niches, thereby re-engaging and reactivating the repair process in the injured distal airway epithelium. Second, resident epithelial stem/progenitor cells in the distal airways may undergo pathological transdifferentiation. It has been established that K13^+^ cells emerge as a transitional phenotype during the differentiation of K5^+^ BCs into CC10^+^ Club cells. We therefore hypothesize that the abnormal accumulation of K13^+^ cells in the epithelium of IPF honeycomb lungs similarly results from an arrested basal-to-Club cell differentiation trajectory, leading to the accumulation of transitional stem cells. This phenomenon may be akin to the aberrant accumulation of K8^+^ cells (K8^+^ ADI) observed in IPF, which is caused by impaired alveolar epithelial differentiation [15,16,31]. In addition, previous studies have shown that mucus secretion levels are significantly higher in the airways of IPF patients compared to healthy lung tissue, with marked upregulation of MUC5AC and MUC5B expression, particularly within honeycomb lesions. In the present study, the marked increase in the number of K13^+^MUC5AC^+^ cells exhibiting adjacent or regionally overlapping expression patterns further suggests that, during their transdifferentiation process, K13^+^ cells not only exhibit a tendency to differentiate toward a proximal pseudostratified epithelial phenotype but also display a more pronounced propensity for secretory cell differentiation than is typically observed in normal proximal airways. This phenomenon implies that, under the pathological conditions of IPF, the distal airway epithelium may undergo a gradual phenotypic transition toward a proximal airway identity.

We further characterized this ectopic cell population at the transcriptomic level. By re-clustering and comparing scRNA sequencing data from distal lung tissues of healthy individuals and IPF patients, we found that the proportions of K13^+^K5^+^ BCs and K13^+^SCGB3A1^+^ Club cells in the distal airways of IPF patients were markedly increased, from 1.8% to 41.5%, and from 10.7% to 31.5%, respectively. In contrast, the proportion of AT2 cells decreased from 87.5% to 27.0%. This shift in cellular composition further suggests that alveolar epithelial cells may undergo cell fate reprogramming toward an airway-like phenotype under the pathological conditions of IPF. Moreover, transcriptomic analysis revealed that K13^+^ BCs in IPF lungs exhibit high expression of a series of risk-associated genes involved in cell growth, differentiation, inflammation, and fibrosis. Among these, LGALS7, which encodes the galectin family, was markedly upregulated. Previous studies have shown that LGALS7 is characteristically enriched in quiescent BCs and in keratinocytes undergoing transitional differentiation states [62,63,64,65]. Overexpression of LGALS7 inhibits the proliferation of multiple epithelial cell types and promotes their differentiation toward a keratinocyte-like phenotype, suggesting that LGALS7 may play a key role in the in vivo migration of K13^+^ BCs, the re-epithelialization of honeycomb cyst walls, and the bronchiolization of alveolar border regions in IPF. In addition, K13^+^ BCs also showed elevated expression of S100A2. As a member of the S100 protein family, S100A2 is primarily involved in various pathological processes, such as fibrosis and tumorigenesis, by regulating epithelial-mesenchymal transition (EMT) [66,67]. Previous studies have demonstrated that S100A2 expression is significantly increased in the lung tissues of IPF patients and promotes EMT through the activation of the Wnt/β-catenin pathway [66], thereby accelerating fibrotic progression. Furthermore, K13^+^ BCs also exhibited high expression of IGFBP6, which plays important roles in regulating cell proliferation, apoptosis, migration, and angiogenesis. In early research reports, elevated IGFBP6 expression was observed in hepatic stellate cells, where it promotes liver fibrosis progression via the TGF-β/SMAD signaling pathway [68,69]. Beyond liver fibrosis, IGFBP6 upregulation has also been observed in multiple other fibrotic disease models, including annulus fibrosus fibrosis of intervertebral disks, skin fibrosis associated with systemic sclerosis, atherosclerotic plaques, and renal fibrosis [70,71,72,73,74]. Similarly, the high expression of IGFBP6 in K13^+^ BCs suggests that this gene may contribute to IPF fibrogenesis through similar mechanisms and further experimental validation is still needed.

To further explore the intercellular crosstalk among distinct cell subsets, we performed CellChat analysis. This revealed that K13^+^ BCs displayed substantially stronger interaction capacities with both epithelial and fibroblast subpopulations relative to their K13 counterparts. In-depth analysis revealed that the retinoic acid (RA) signaling pathway plays a central role in the communication between K13^+^ BCs and surrounding cells. Specifically, K13^+^ BCs participate in multiple inter-epithelial interactions primarily through RA receptors expressed on their surface. The major ligands involved in this signaling axis are provided by ALDH1A1 and ALDH1A3, while the corresponding receptors on K13^+^ BCs include RXRA in complex with CRABP2, as well as RARG associated with CRABP2. Both RARG and RXRA are RA receptor subtypes broadly expressed in the airway epithelium, and many of the essential physiological functions of retinol (vitamin A) are mediated through these receptor pathways [75,76,77]. As a key regulator of airway development, retinol has been shown to be essential for maintaining airway epithelial integrity; accordingly, stable expression of RA receptors in normal airways contributes to sustaining epithelial barrier homeostasis [78]. Furthermore, RA receptor agonists have been reported to alleviate inflammation, improve hypoxic microenvironments, and reduce fibrosis severity, in part by downregulating TGF-β expression and inhibiting EMT [79]. Taken together, RA receptor signaling can be viewed as an intrinsically protective pathway in the airway. Based on these findings, we propose that this specific pathway may play a significant regulatory role in the aberrant differentiation of K13^+^ BCs toward a proximal airway epithelial phenotype in IPF honeycomb lungs. This raises the possibility that such abnormal differentiation represents a pathological repair response of lung epithelium under persistent exposure to multiple injurious stimuli, including hypoxia and inflammation, in the context of IPF.

## 5. Conclusions

In this study, we revealed the ectopic expression of K13^+^ epithelial cells in IPF and explored its pathological implications. Spatial analysis along the proximal-distal airway axis demonstrated that K13 is heterogeneously and prominently upregulated in IPF lung tissues, with the highest proportion of K13^+^ cells localized within subpleural honeycomb cysts, suggesting a proximalization of the distal airway epithelium. Multiplex immunofluorescence staining confirmed that the epithelium lining honeycomb cysts exhibits a proximal pseudostratified epithelial-like phenotype, and residual epithelial cells showed downregulation of SOX2 with co-expression of K13. scRNA-seq showed a marked expansion of K13^+^ cell subpopulations and enriched pro-fibrotic genes, as well as pathways related to RNA processing and stress responses, while exhibiting a significantly enhanced senescence-associated phenotype. CellChat analysis further uncovered heightened intercellular signaling interactions involving K13^+^ BCs and AT2 cells with other epithelial subsets and with HAS1^high^ fibroblasts, suggesting that K13^+^ cells may serve as a critical modulating factor of inflammatory and stromal activation signals. Taken together, several key findings in our study, including the ectopic expression of K13, the phenotypic shift toward a proximal airway epithelial identity, the pro-fibrotic transcriptional signature, and the enhanced fibrosis-related signaling crosstalk, all point to a pathological proximalization of the distal airway epithelium in IPF, which may represent a potential mechanism driving bronchiolization and contributing to disease progression.

## Data Availability

The original contributions presented in this study are included in the article material. Further inquiries can be directed to the corresponding author.

## Supplementary Materials

The following supporting information can be downloaded at: https://www.mdpi.com/article/10.3390/biomedicines14030728/s1; Figure S1: HE and K13 immunofluorescence staining of the bronchial tree from normal lung tissue; Figure S2: Panoramic view of K13 distribution in the airway epithelium of IPF, and K13 immunofluorescence staining of ex vivo cultured cells; Figure S3: Reclustering of the scRNA-seq data revealed the coexistence of multiple K13^+^ airway epithelial cell states (GSE135893); Figure S4: Quality control of single-cell analysis of K13^+^ cells (GSE135893); Figure S5: Reclustering of the scRNA-seq data revealed the coexistence of multiple K13^+^ airway epithelial cell states (GSE227136); Figure S6: Specific ligand-receptor interactions in the signaling pathways emitted from K13^+^ and K13^−^ BC and AT2 to other airway epithelial cell subtypes (GSE135893); Figure S7: RA, LAMININ, and THBS signaling chord diagrams in the cell communication between K13^+^ and K13^−^ BC and AT2 cells and epithelial cell subpopulations (GSE135893); Figure S8: Specific ligand-receptor interactions in the cell communication between K13^+^ and K13^−^ BC and AT2 cells with fibroblast subpopulations (GSE135893); Table S1: Clinical sample information used for pathological tissue and cellular studies.

## Nonstandard Abbreviations and Acronyms

**K13**	Cytokeratin 13, KRT13
**K5**	Cytokeratin 5, KRT5
**K17**	Cytokeratin 17, KRT17
**K8**	Cytokeratin 8, KRT8
**IPF**	Idiopathic pulmonary fibrosis
**BC**	Basal cell
**BALF**	Bronchoalveolar lavage fluid
**SD**	Standard deviation
**HRCT**	High-resolution computed tomography
**MDCT**	Multidetector CT
**scRNA-seq**	Single-cell RNA sequencing
**SASP**	Senescence-associated secretory phenotype
**ECM**	Extracellular matrix
**SPB**	Secretory primed BC
**AT1**	Alveolar type 1
**AT2**	Alveolar type 2
**ADI**	Alveolar differentiation intermediate
**AREG**	Amphiregulin
**HBEGF**	Heparin-binding EGF-like growth factor
**ITGB6**	Integrin β6
**GEO**	Gene Expression Omnibus
**RA**	Retinoic acid
**GO**	Gene ontology
**EMT**	Epithelial-mesenchymal transition
**DAPI**	4,6-diamidino-2-phenylindole
**PCA**	Principal component analysis
