# Supplementary material for: Spatial Distribution of K13-Positive Airway Epithelial Cells in Idiopathic Pulmonary Fibrosis
Source: Biomedicines. 2026 Mar 23;14(3):728. doi: 10.3390/biomedicines14030728 (PMC13024621; doi:10.3390/biomedicines14030728)
Supplement: Supplementary file 1 [file biomedicines-14-00728-s001.zip › Supplementary information-260311.pdf]

INVENTORY OF ALL SUPPLEMENTAL ITEMS

Supplementary Figure S1 HE and K13 immunofluorescence staining of the bronchial tree from normal lung tissue.

Supplementary Figure S2 Panoramic view of K13 distribution in the airway epithelium of IPF, and K13 immunofluorescence staining of ex vivo cultured cells.

Supplementary Figure S3. Reclustering of the scRNA-seq data revealed the coexistence of multiple K13$^+$ airway epithelial cell states (GSE135893).

Supplementary Figure S4. Quality control of single-cell analysis of K13$^+$ cells (GSE135893).

Supplementary Figure S5. Reclustering of the scRNA-seq data revealed the coexistence of multiple K13$^+$ airway epithelial cell states (GSE227136).

Supplementary Figure S6. Specific ligand-receptor interactions in the signaling pathways emitted from K13$^+$ and K13$^-$ BC and AT2 to other airway epithelial cell subtypes (GSE135893).

Supplementary Figure S7.RA, LAMININ, and THBS signaling chord diagrams in the cell communication between K13$^+$ and K13$^-$ BC and AT2 cells and epithelial cell subpopulations (GSE135893).

Supplementary Figure S8. Specific ligand-receptor interactions in the cell communication between K13$^+$ and K13$^-$ BC and AT2 cells with fibroblast subpopulations (GSE135893).

Table S1. Clinical sample information used for pathological tissue and cellular studies.

Supplementary Figure S1. HE and K13 immunofluorescence staining of the bronchial tree from normal lung tissue.

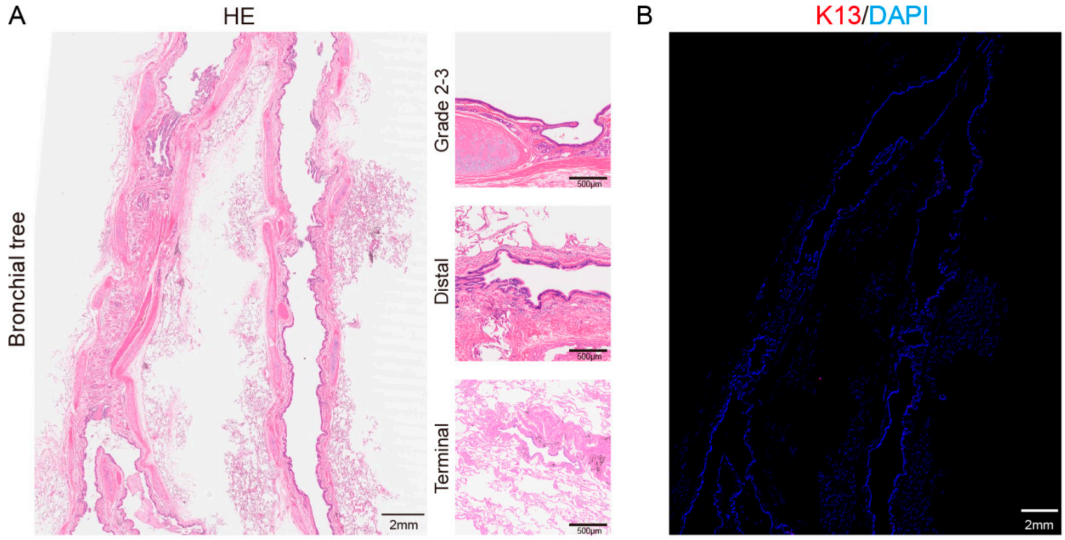

(A) HE staining of the bronchial tree from normal control lung sample, including Grades 5-9 bronchial tree (left), Grade 2-3 proximal airway (upper right), distal lung (middle right), and terminal regions (lower right).

(B) Panoramic immunofluorescence staining of K13 in the bronchial tree from healthy lung sample.

Supplementary Figure S2. Panoramic view of K13 distribution in the airway epithelium of IPF, and K13 immunofluorescence staining of ex vivo cultured cells.

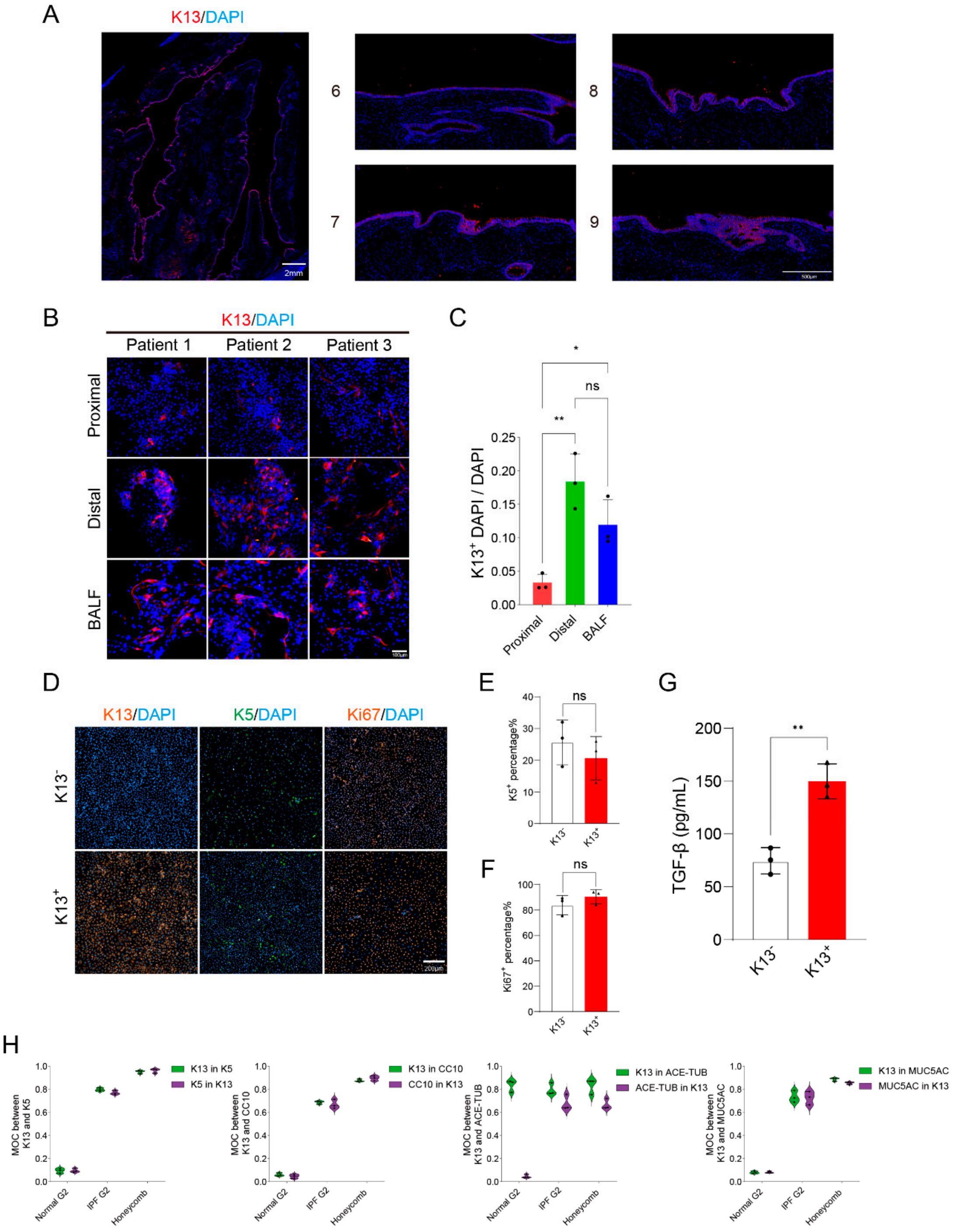

(A) Bronchial tree section from IPF human lungs stained for K13 (left), with high

magnification images of Grade 6-9 airways (right).

(B) TGF-β levels in the culture supernatants of $K13^+$ and $K13^-$ cells from Figure D were quantified by ELISA, followed by statistical analysis (N=3 patients)**.** ** indicates P < 0.01.

(C-D) K13 immunofluorescence staining images of ex vivo cultured airway epithelial cells from different sampling sites (proximal, distal, BALF) of IPF patients (N=3), with statistical analysis of the proportion of $K13^+$ cells. One-way ANOVA statistical analysis means ± SD. * indicates P < 0.05, ** indicates P < 0.01, while NS denotes no statistical significance (P > 0.05).

(E-G) Immunofluorescence staining was performed following in vitro culture of $K13^+$ and $K13^-$ cells sorted from BALF of IPF patients. Cells were stained for K13(orange), Ki67 (orange) and K5 (green) (D), along with corresponding statistical analysis of $K5^+$ cell and $Ki67^+$ cell proportions (N=3 patients) (E-F). ns denotes no statistical significance (P > 0.05).

(H) Mander's overlap coefficients (MOC) for Panel A were calculated using Image J. Each dot represents the mean value of all Z-stack images from a single region of interest.

Supplementary Figure S3. Reclustering of the scRNA-seq data revealed the coexistence of multiple K13^{+} airway epithelial cell states (GSE135893).

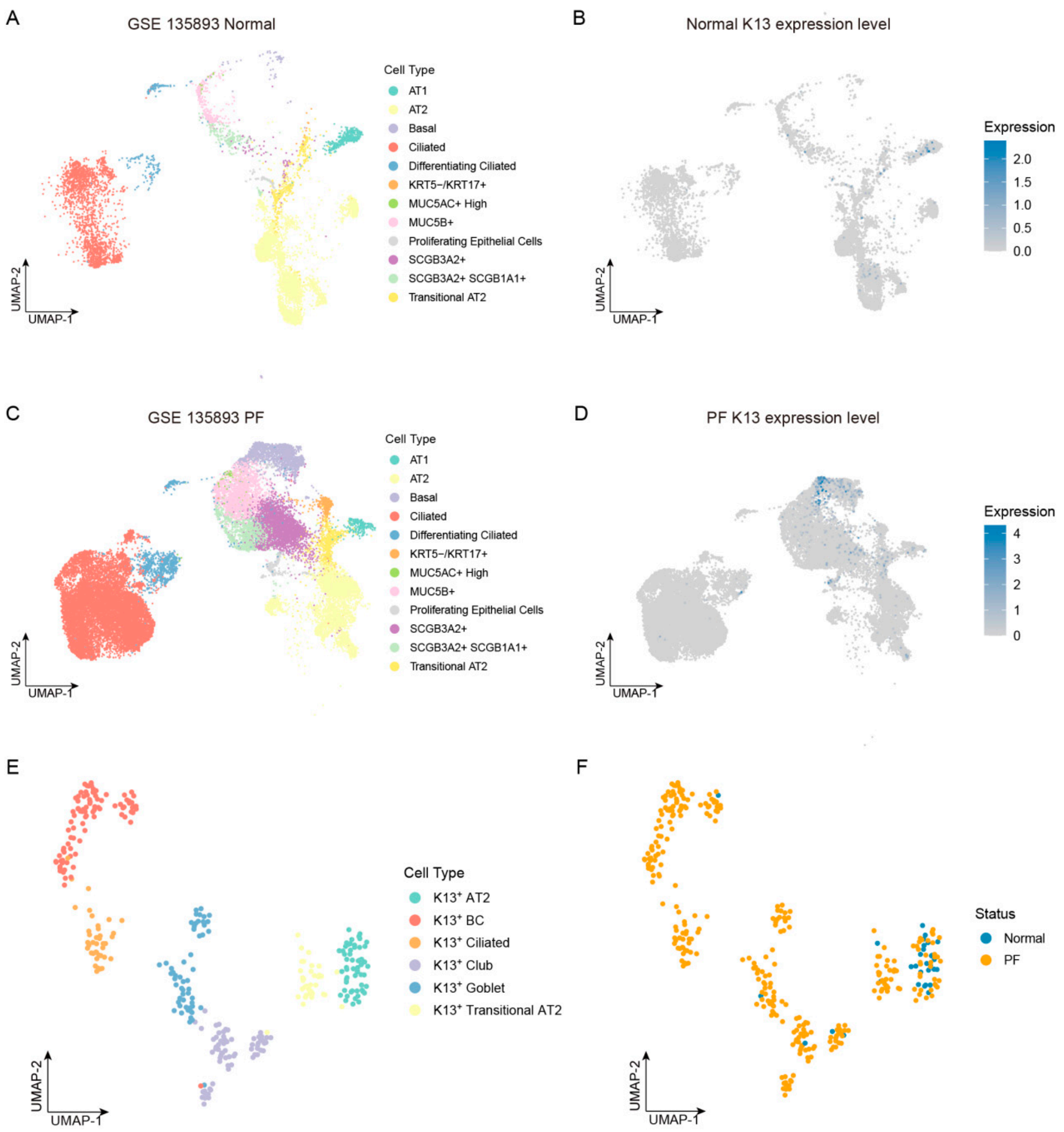

(A-D) Unsupervised clustering was performed on epithelial cells from GSE135893 grouped into normal and PF cohorts, and K13 expression was projected onto the UMAP embedding.

(E-F) K13^{+} cells were subsetted for dedicated UMAP-based reclustering, revealing that the K13^{+} compartment encompasses multiple epithelial identities.

Supplementary Figure S4. Quality control of single-cell analysis of K13⁺ cells (GSE135893).

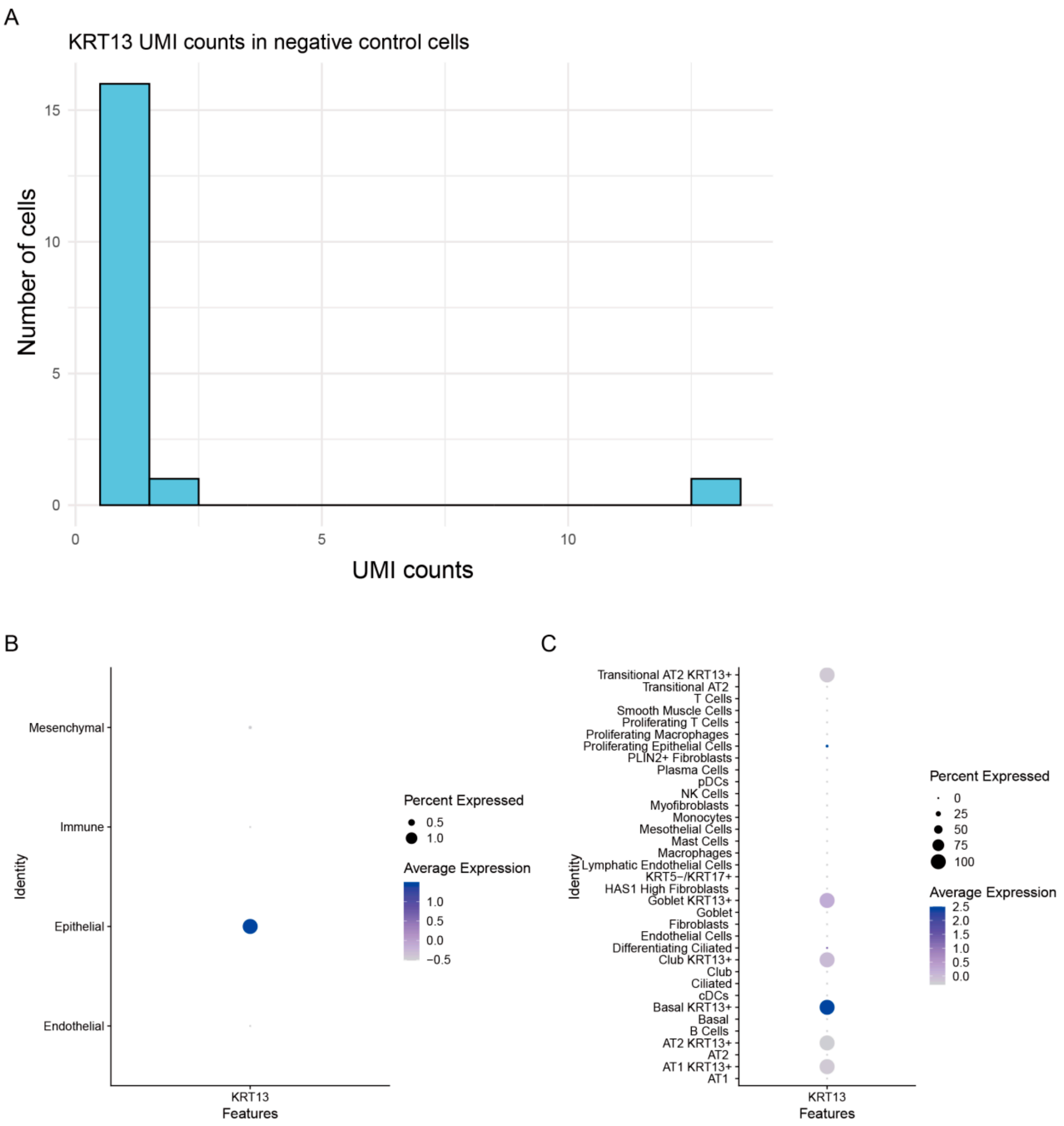

(A) UMI distribution of K13 across immune cell populations.

(B-C) Bubble plot for K13-positive cell types.

Supplementary Figure S5. Reclustering of the scRNA-seq data revealed the coexistence of multiple K13^{+} airway epithelial cell states (GSE227136).

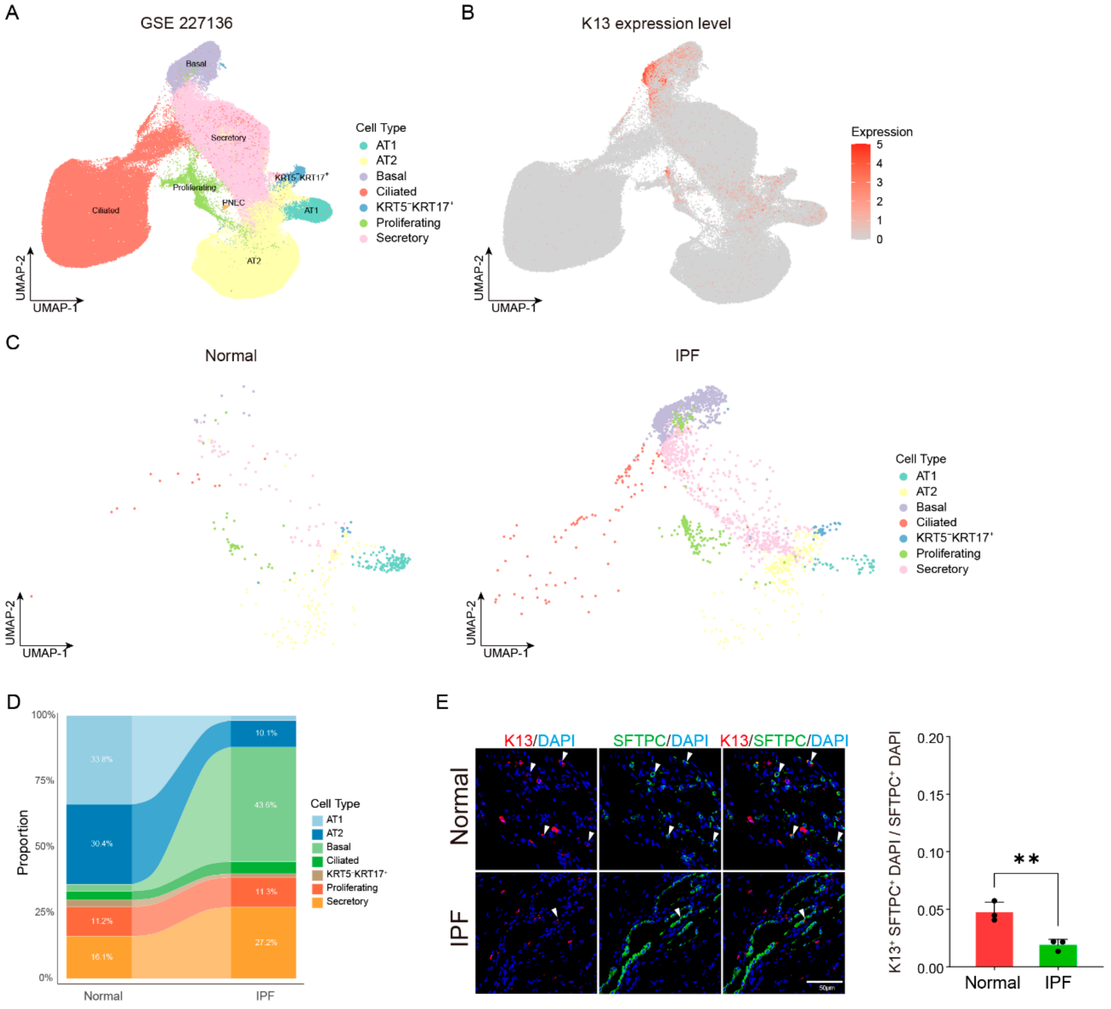

(A-B) Unsupervised clustering was performed on epithelial cells from GSE227136 grouped into normal and PF cohorts, and K13 expression was projected onto the UMAP embedding.

(C-D) Re-clustering of the K13^{+} epithelial cell population from this dataset using UMAP (C), with quantification of the proportion of each cluster (D).

(E) Co-staining of K13 (red) and alveolar type II cell markers (SFTPC) (green) in the normal and IPF lung (N=3 patients).

Supplementary Figure S6. Specific ligand-receptor interactions in the signaling pathways emitted from K13⁺ and K13⁻ BC and AT2 to other airway epithelial cell subtypes (GSE135893).

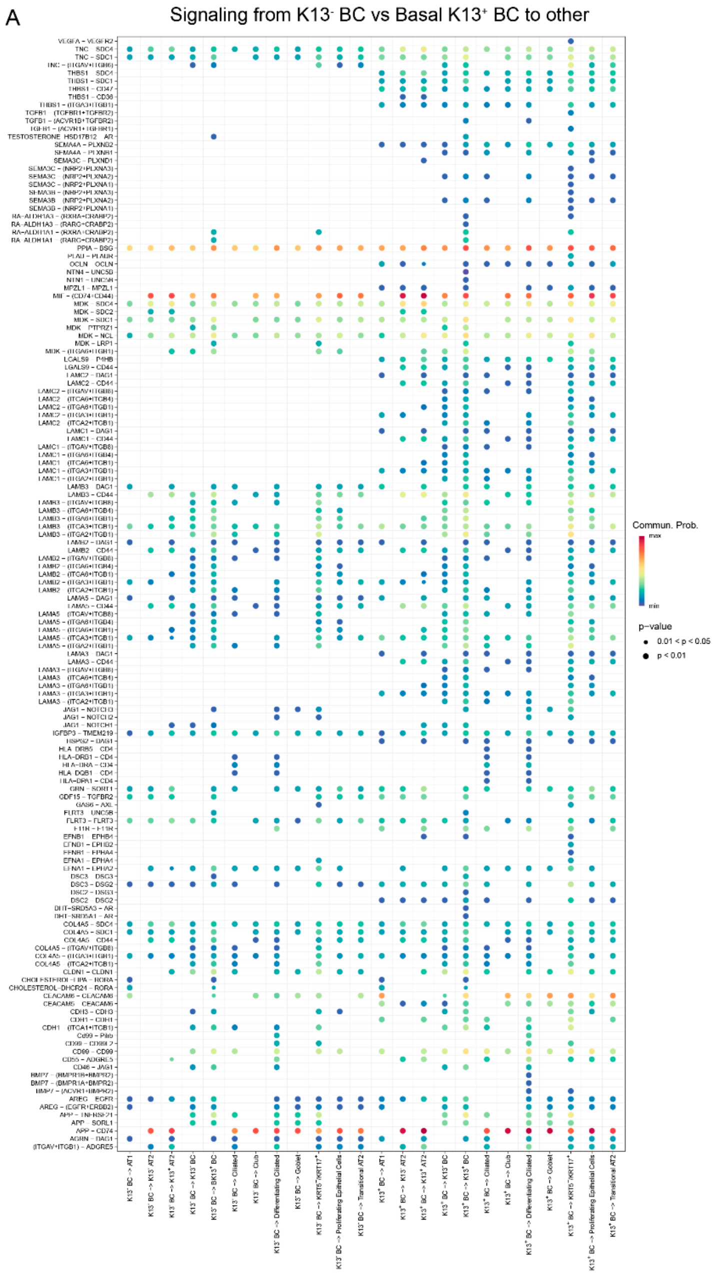

B

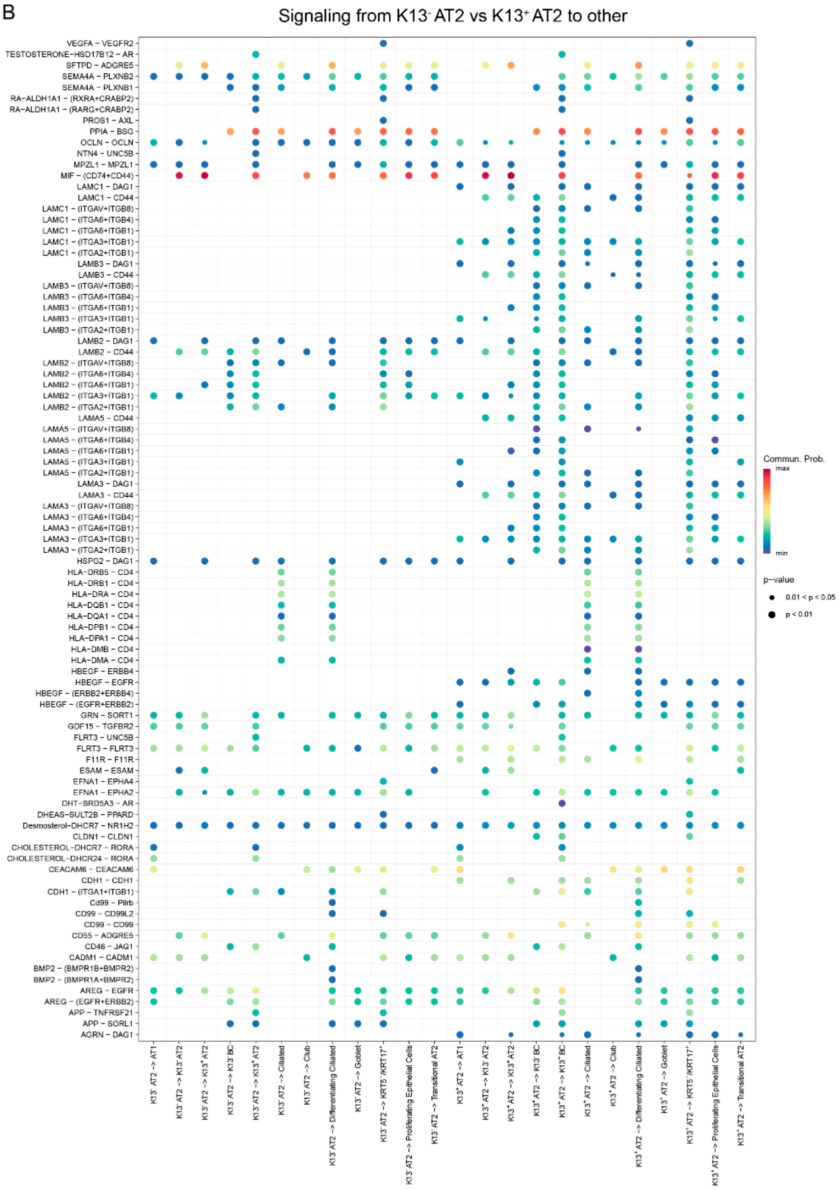

Comparison of all significant ligand-receptor pathways sending signals from K13^{+} and K13^{-} BC **(A)**, as well as from K13^{+} and K13^{-} AT2 **(B)** to airway epithelial cell subtypes.

Supplementary Figure S7. RA, LAMININ, and THBS signaling chord diagrams in the cell communication between K13^{+} and K13^{-} BC and AT2 cells and epithelial cell subpopulations (GSE135893).

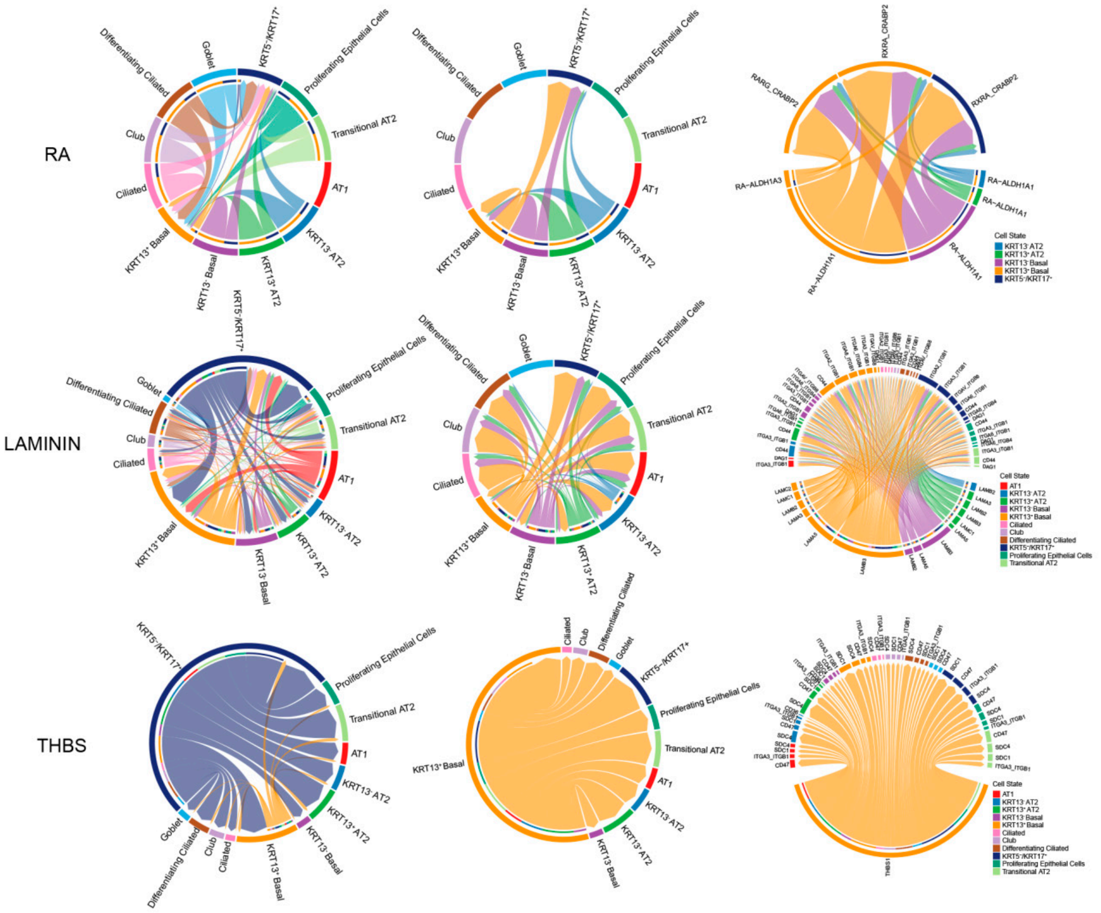

Illustrate the intercellular signaling interactions between K13^{+} BC and K13^{+} AT2 cells and other epithelial cells through the RA, LAMININ, THBS signaling pathways. Left to right panels represent overall cluster communication, isolated communication when K13^{+} BC and AT2 act as ligand cells, and detailed ligand–receptor communication within each pathway.

Supplementary Figure S8. Specific ligand-receptor interactions in the cell communication between K13$^+$ and K13$^-$ BC and AT2 cells with fibroblast subpopulations (GSE135893).

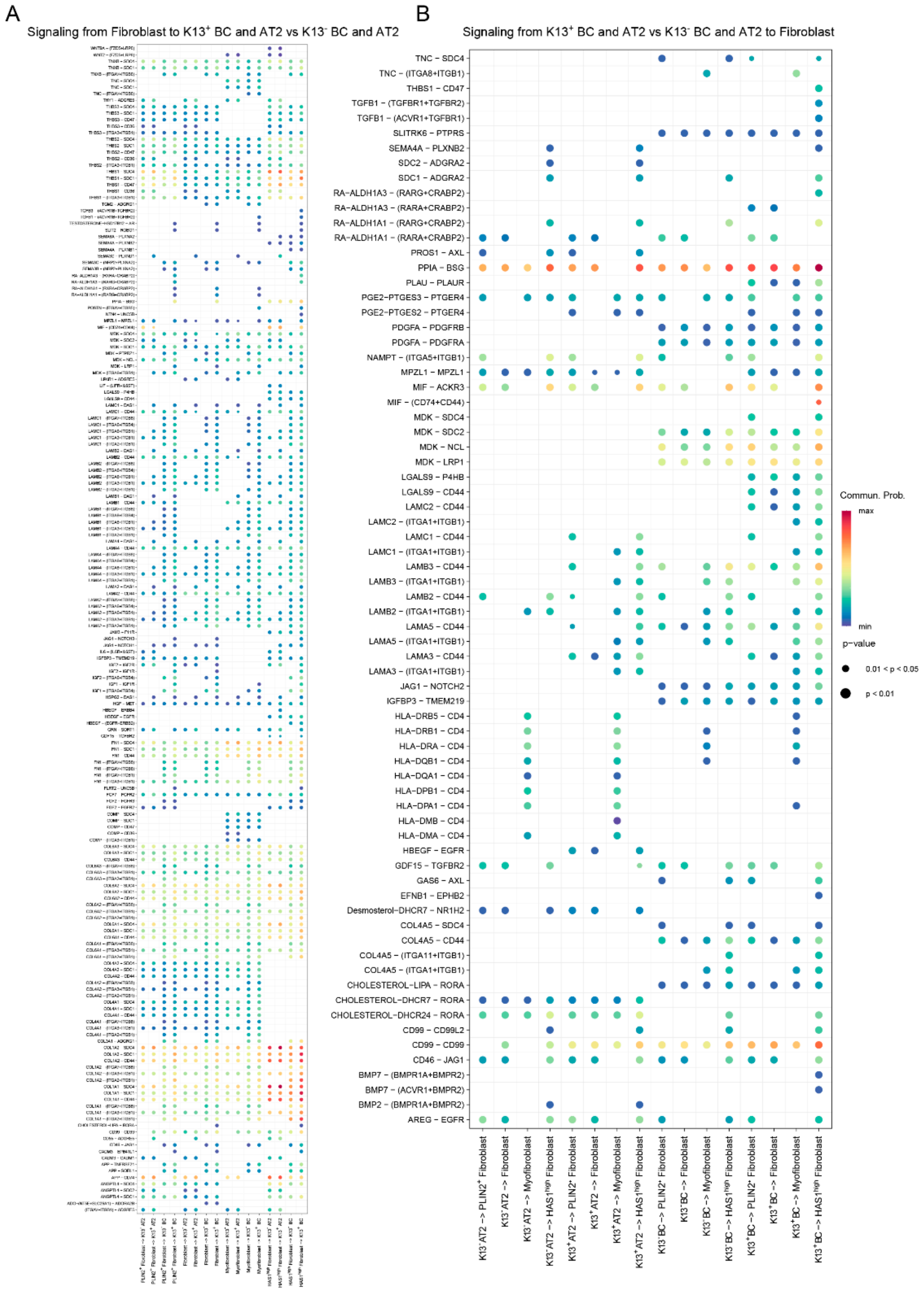

(A) All significant ligand-receptor pathways sent from fibroblast subpopulations to K13$^+$ and K13$^-$ BC and AT2 cells.

(B) All significant ligand-receptor pathways sent from $K13^+$ and $K13^-$ BC and AT2 cells to fibroblast subpopulations.

Table S1: Clinical sample information used for pathological tissue and cellular studies

Sample type	Patient ID	Sex	Age,yr	Former smoker	Severity stage
Lung tissue	Ctrl1	Male	58	Yes	None
	Ctrl2	Female	71	No	None
	Ctrl3	Female	48	No	None
	Ctrl4	Female	51	No	None
	IPF1	Male	50	No	Severe
	IPF2	Male	59	No	Severe
	IPF3	Male	72	Yes	Severe
	IPF4	Female	48	No	Severe
Primary cells	IPF5	Female	66	No	Severe
	IPF6	Female	75	Yes	Severe
	IPF7	Male	64	Yes	Severe
